# Supplementary material for: Epigenetic and genetic components of height regulation
Source: Nat Commun. 2016 Nov 16;7:13490. doi: 10.1038/ncomms13490 (PMC5116096; doi:10.1038/ncomms13490)
Supplement: Supplementary Information — Supplementary Figure 1-19, Supplementary Table 1-9, Supplementary Discussion, and Supplementary References [file ncomms13490-s1.pdf]

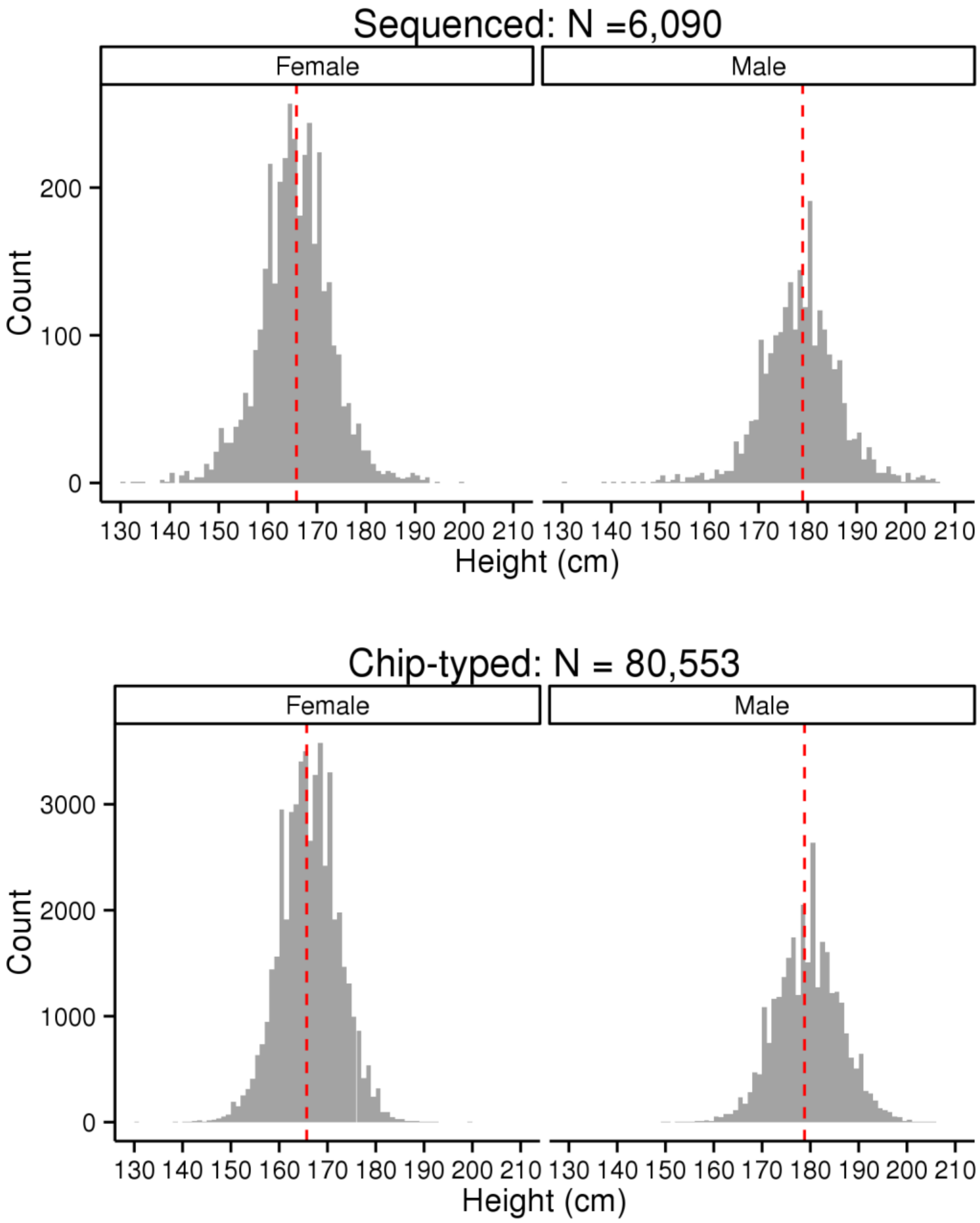


**Supplementary Fig. 1.**

Histograms showing the distribution of raw height values split by sex of a) sequenced with height-measurements (N=6,090) and b) chip-typed individuals with height-measurements (N=80,546). Red dotted lines correspond to mean height of chip-typed individuals with adult height measurements: Female=165.6 cm, Male=178.8 cm.

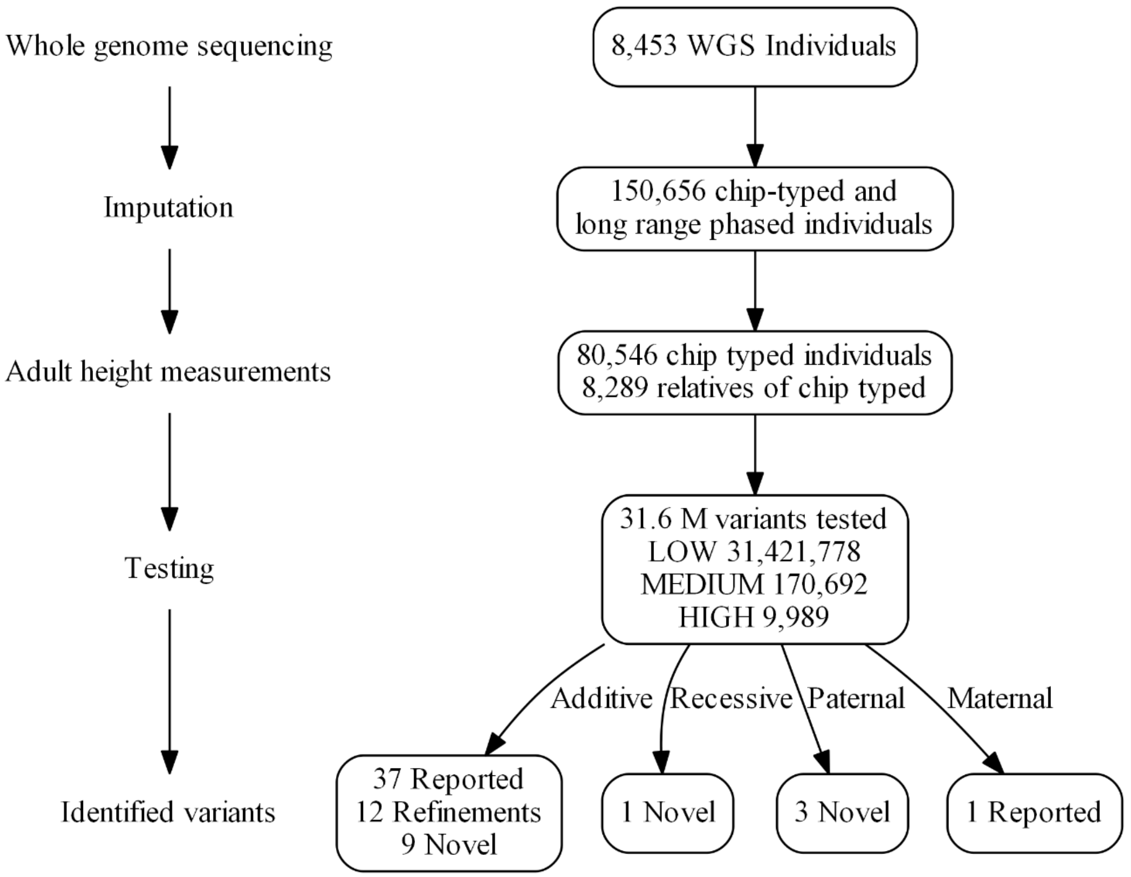


**Supplementary Fig. 2.**

Flowchart of the genome-wide association study design.

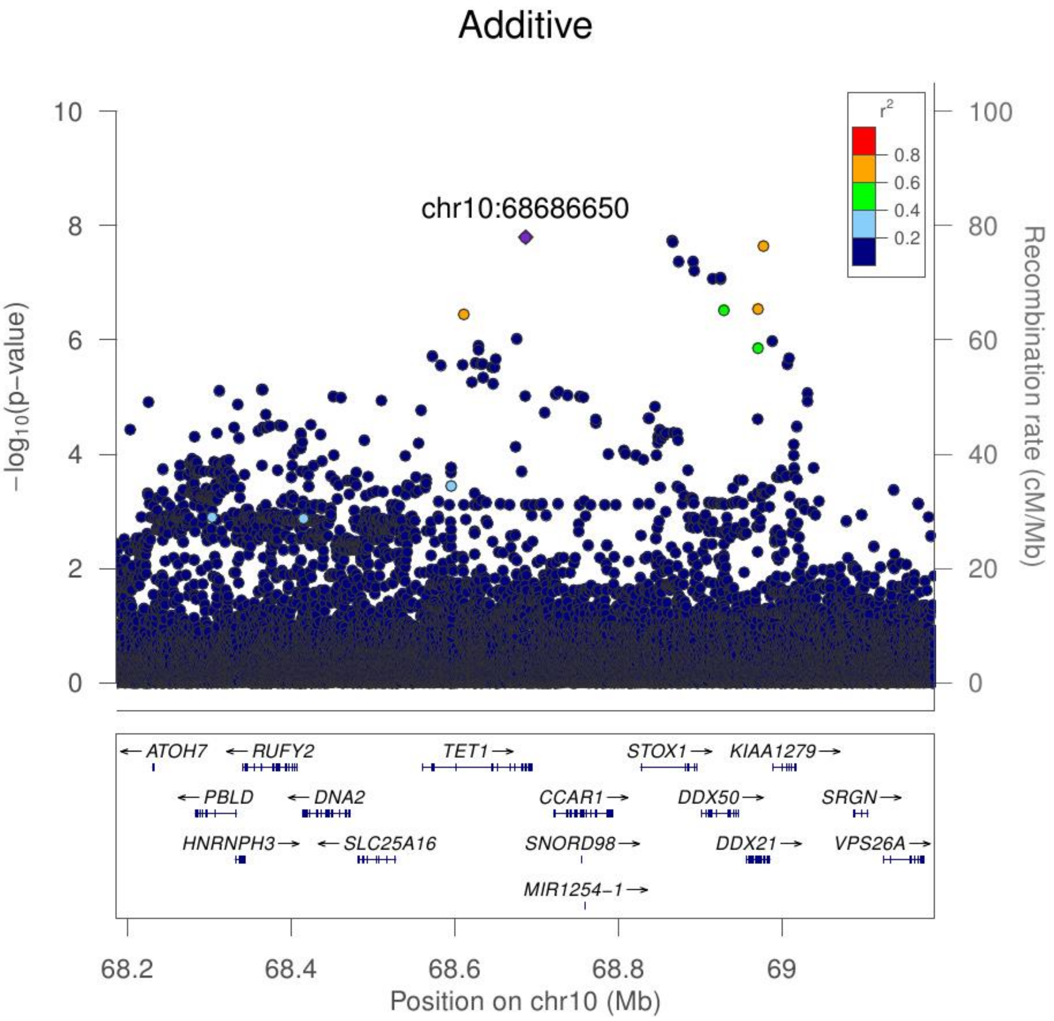


**Supplementary Fig. 3.**

Locus plot depicting variants at the *TET1* locus (hg38) associating with adult height. The leading variant, rs558226101[T], is labelled as a diamond and shown in purple, other variants are colored according to correlation ($r^2$) with the leading marker (legend at top-right). $-\log_{10}$ *P*-values are shown along the left y-axis and correspond to the variants depicted in the plot. The right y-axis shows calculated recombination rates at the chromosomal location, plotted as a solid grey line.

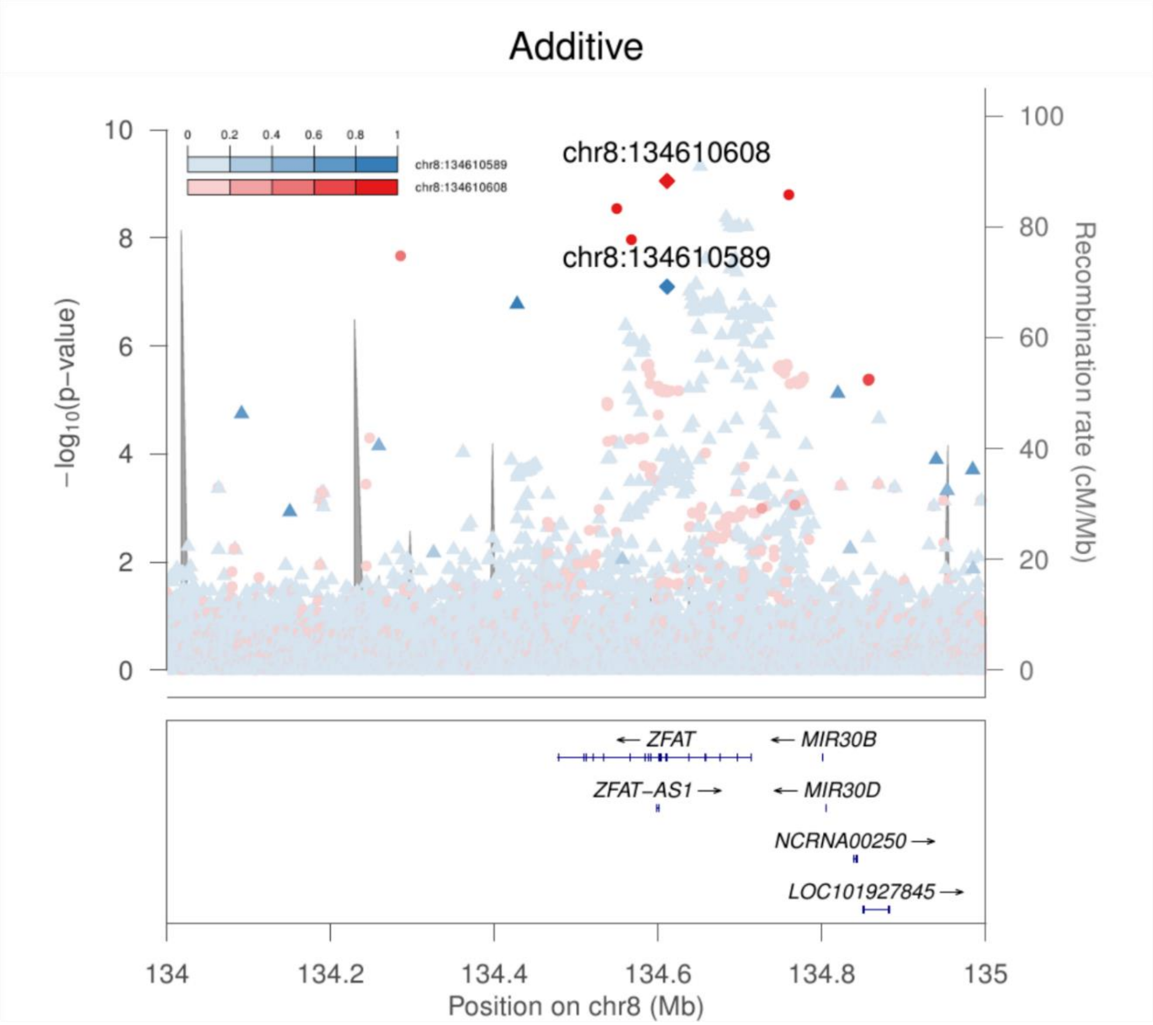


**Supplementary Fig. 4.**

Locus plots depicting variants at the *ZFAT* locus (hg38) associating with adult height. The leading variants of two independent signals, rs75596750[A] and rs201828593[G], are labelled as diamonds and shown in red and blue, respectively. Other variants are colored according to correlation ($r^2$) with their leading marker (darker colors indicating stronger correlation). -$\log_{10}$ P-values are shown along the left y-axis and correspond to the variants depicted in the plot. The right y-axis shows calculated recombination rates at the chromosomal location, plotted as a solid grey line.

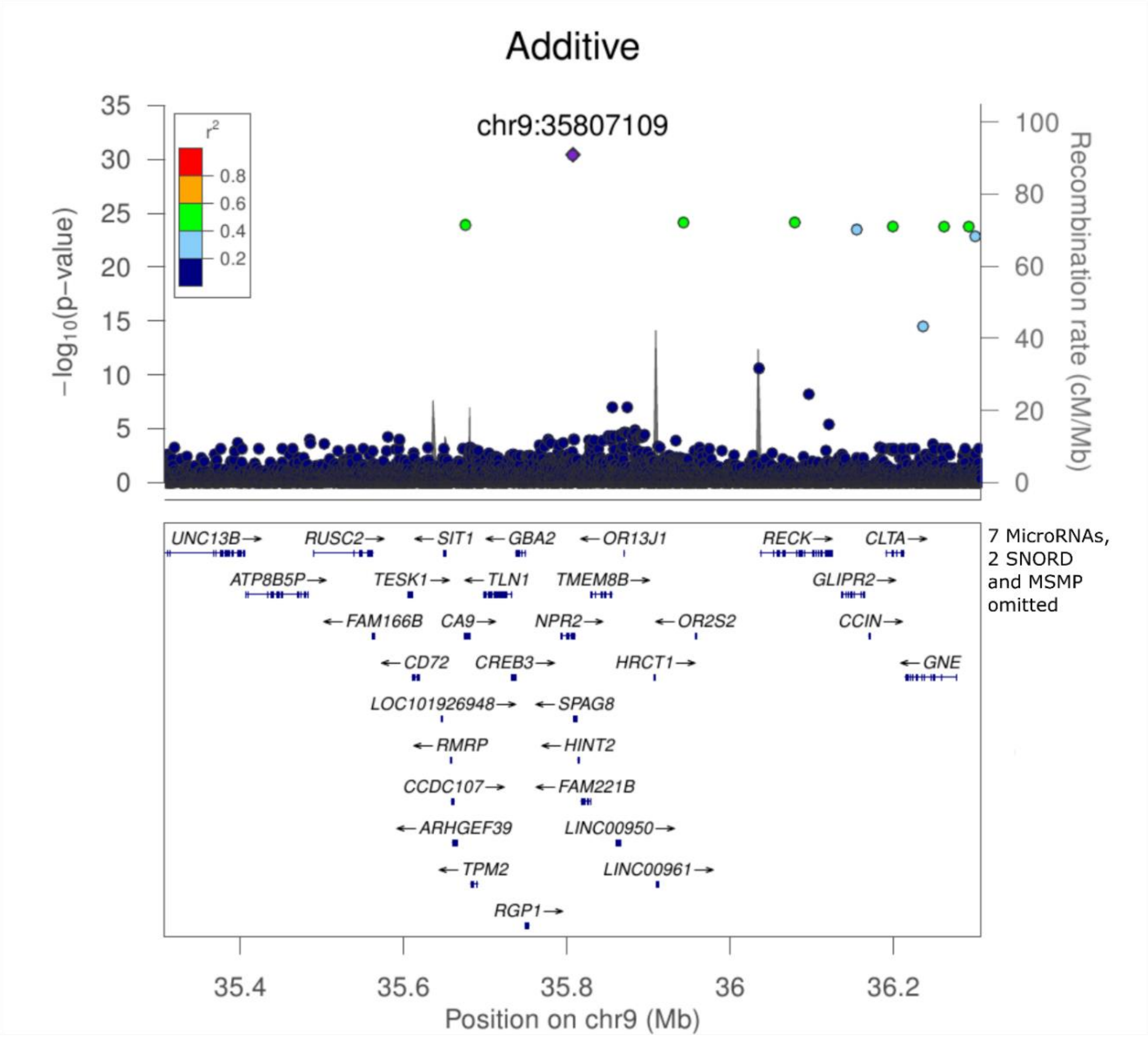


**Supplementary Fig. 5.**

Locus plot depicting variants at the *NPR2* locus (hg38) associating with adult height. The leading variant, chr9:35807109[T], is labelled as a diamond and shown in purple, other variants are colored according to correlation ($r^2$) with the leading marker (legend at top-left). -$\log_{10}$ P-values are shown along the left y-axis and correspond to the variants depicted in the plot. The right y-axis shows calculated recombination rates at the chromosomal location, plotted as a solid grey line.

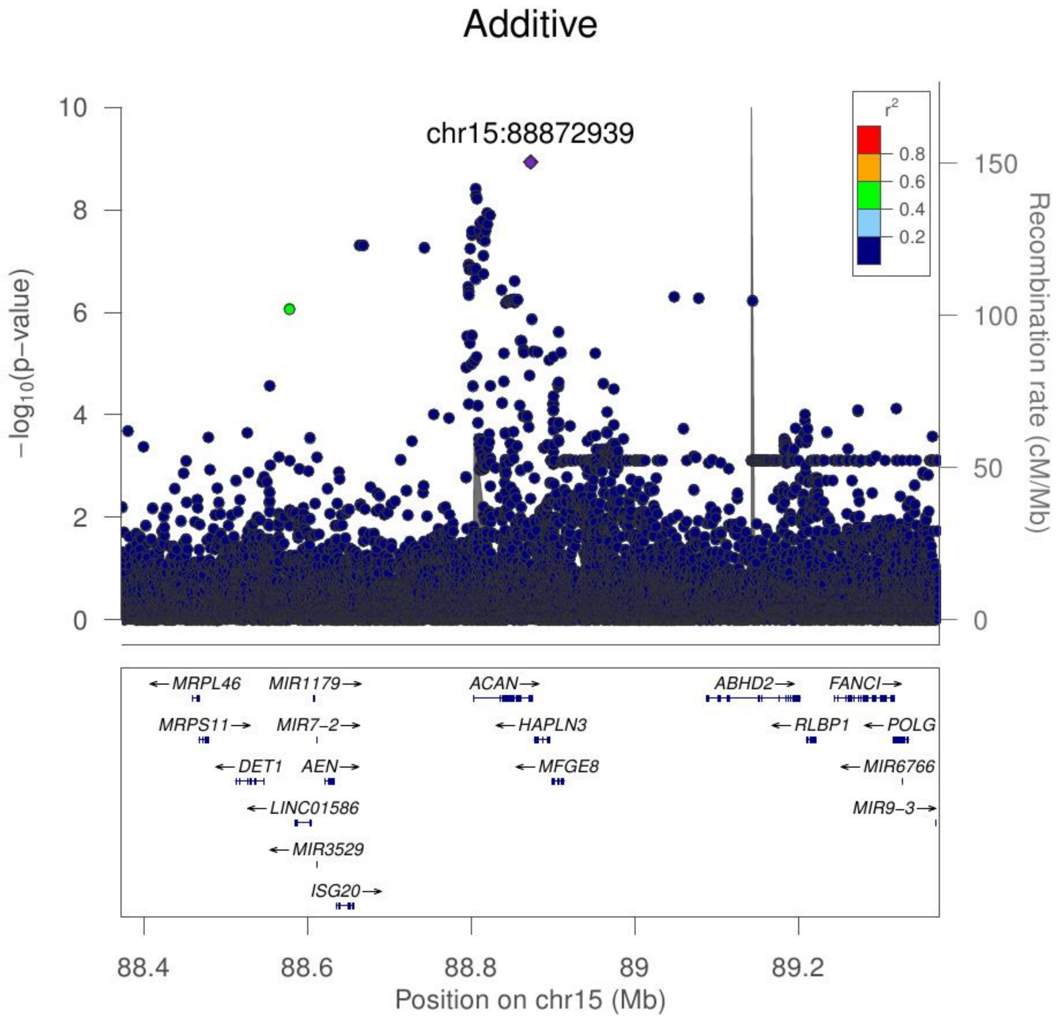


**Supplementary Fig. 6.**

Locus plot depicting variants at the *ACAN* locus (hg38) associating with adult height. The leading variant, chr15:88872939[A], is labelled as a diamond and shown in purple, other variants are colored according to correlation ($r^2$) with the leading marker (legend at top-right). -$\log_{10}$ P-values are shown along the left y-axis and correspond to the variants depicted in the plot. The right y-axis shows calculated recombination rates at the chromosomal location, plotted as a solid grey line.

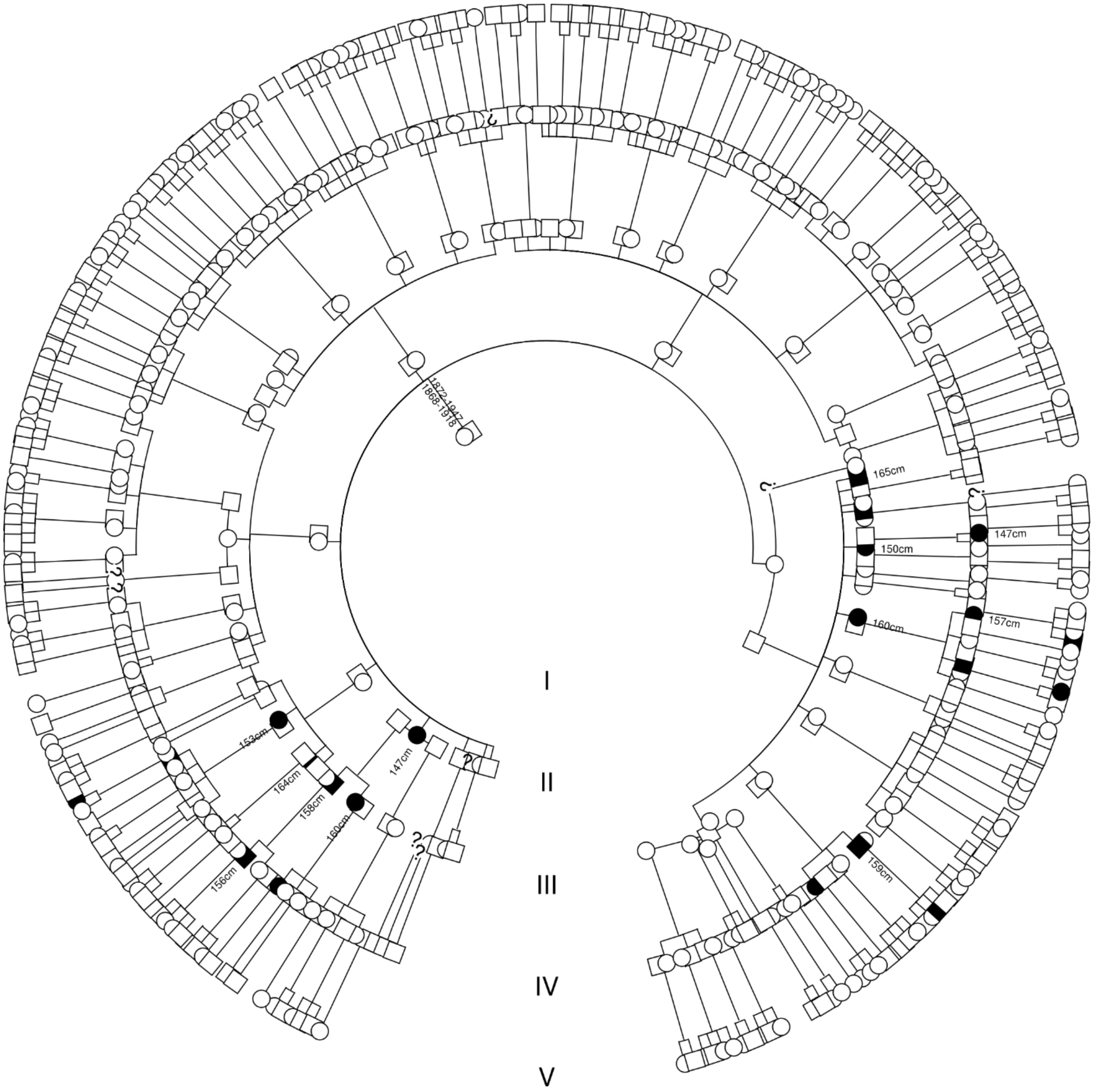


**Supplementary Fig. 7.**

Pedigree depicting family where the *ACAN* variant chr15:88872939[A] was observed. Confirmed carriers are shown in black, and their heights are given in centimeters when available.

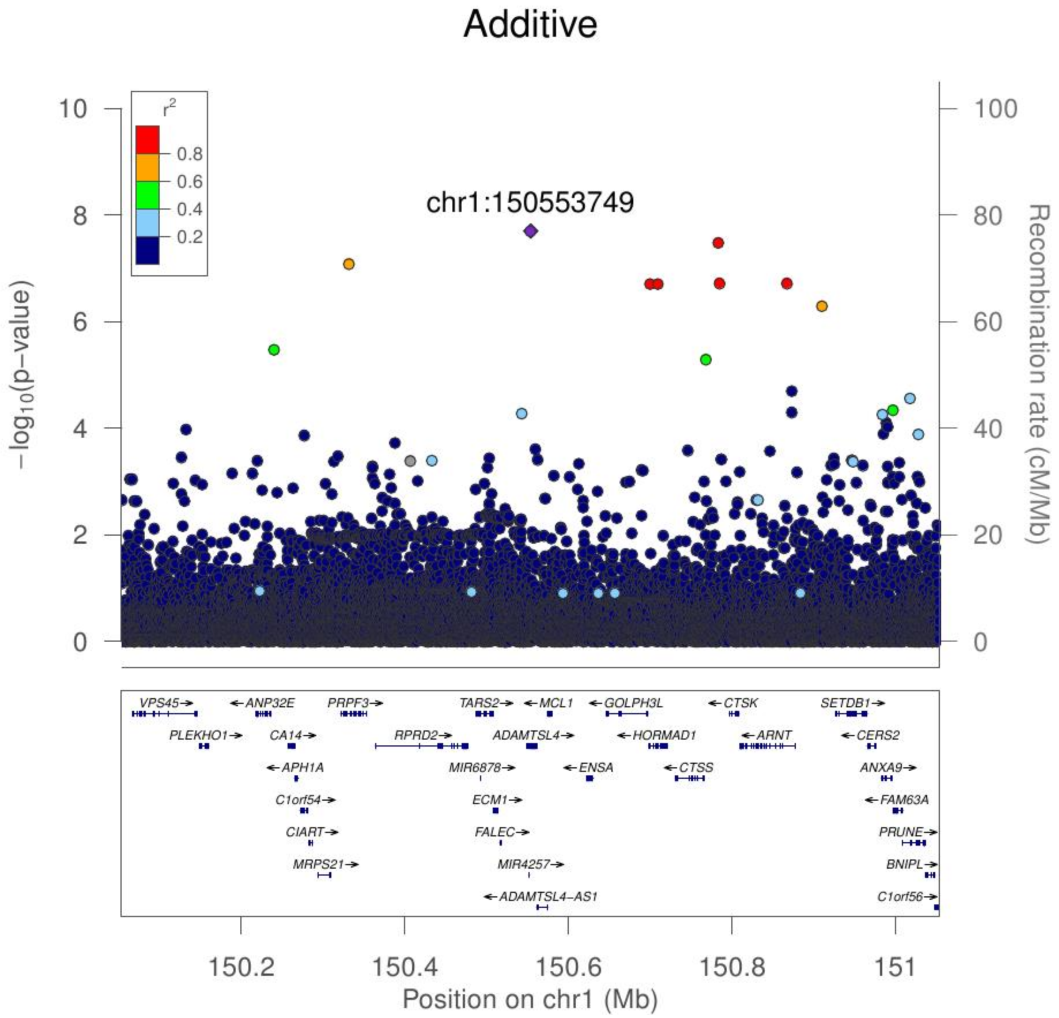


**Supplementary Fig. 8.**

Locus plot depicting variants at the *ADAMTSL4* locus (hg38) associating with adult height. chr1:150553749[C] is labelled as a diamond and shown in purple, and other variants are colored according to correlation ($r^2$) with that marker (legend at top-left). -$\log_{10}$ P-values are shown along the left y-axis and correspond to the variants depicted in the plot. The right y-axis shows calculated recombination rates at the chromosomal location, plotted as a solid grey line.

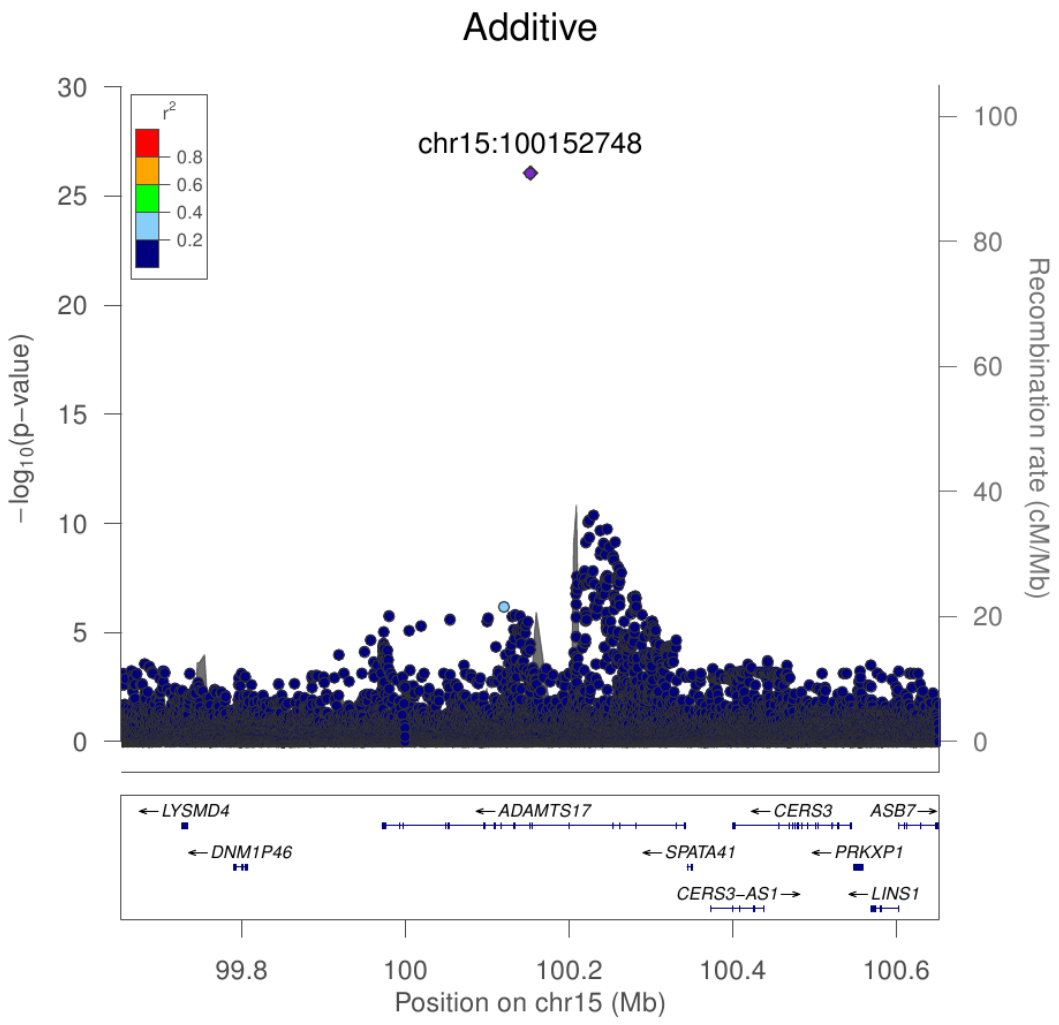


**Supplementary Fig. 9.**

Locus plot depicting variants at the *ADAMTS17* locus associating with adult height. The leading variant, rs72755233[A], is labelled as a diamond and shown in purple, other variants are colored according to correlation ($r^2$) with the leading marker (legend at top-left). -$\log_{10}$ *P*-values are shown along the left y-axis and correspond to the variants depicted in the plot. The right y-axis shows calculated recombination rates at the chromosomal location, plotted as a solid grey line.

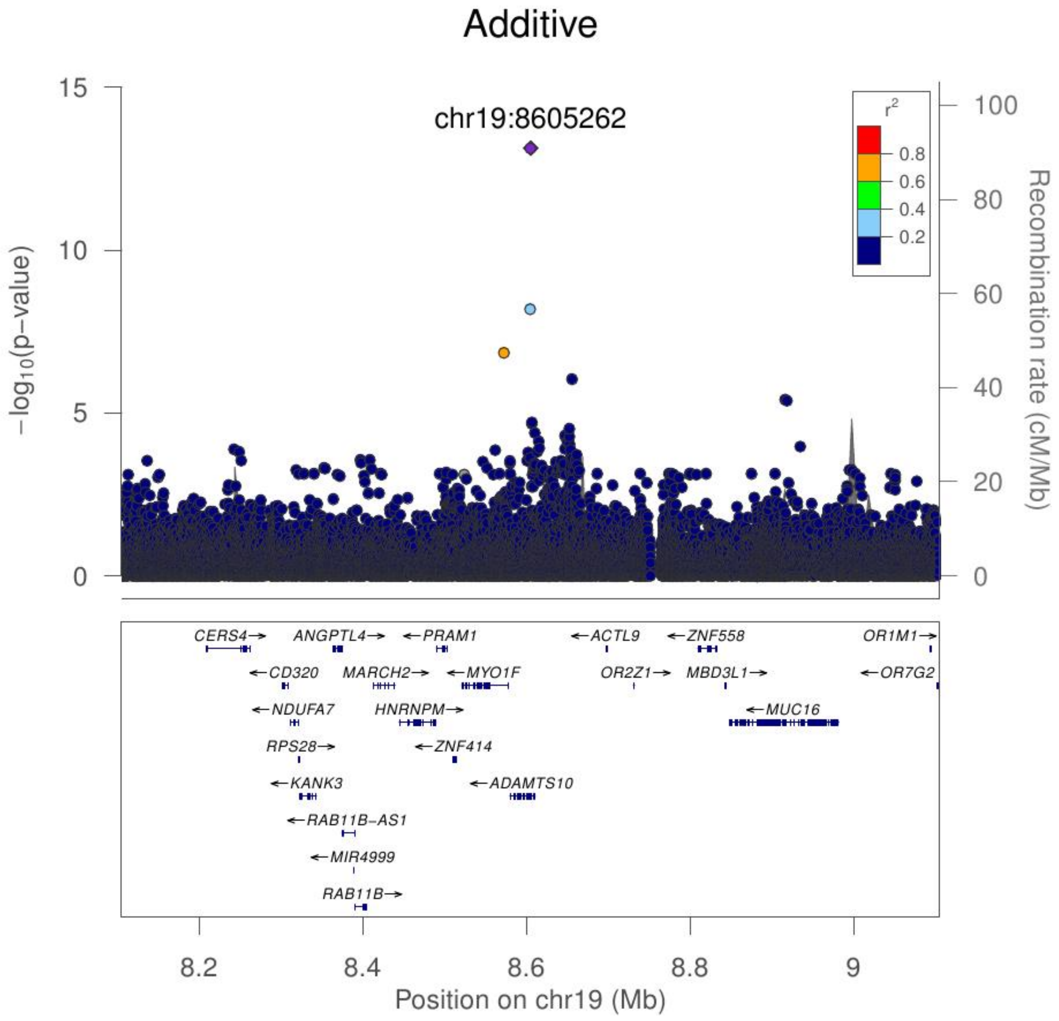


**Supplementary Fig. 10.**

Locus plot depicting variants at the *ADAMTS10* locus associating with adult height. The leading variant, rs62621197[T], is labelled as a diamond and shown in purple, other variants are colored according to correlation ($r^2$) with the leading marker (legend at top-right). -$\log_{10}$ P-values are shown along the left y-axis and correspond to the variants depicted in the plot. The right y-axis shows calculated recombination rates at the chromosomal location, plotted as a solid grey line.

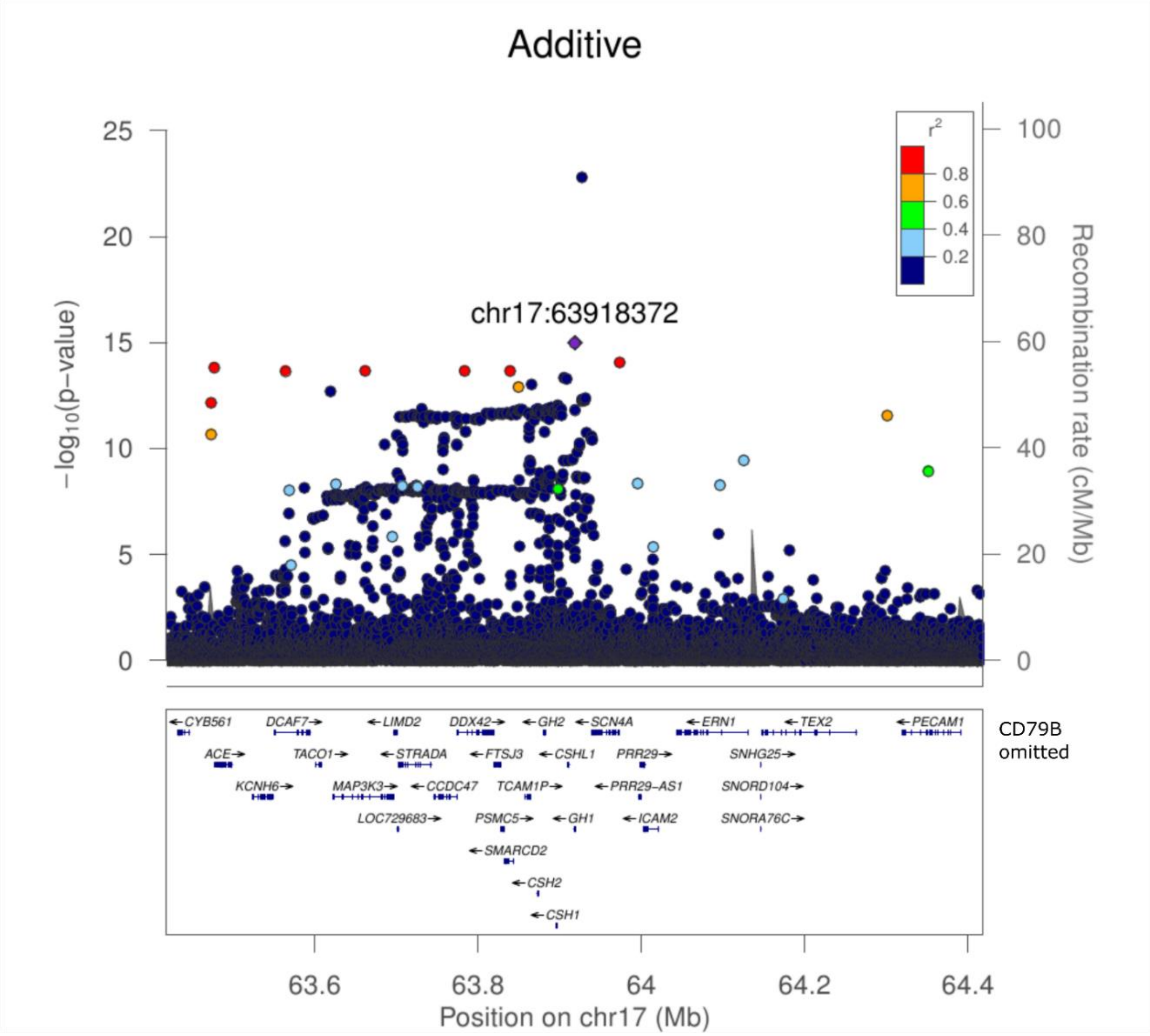


**Supplementary Fig. 11.**

Locus plot depicting variants at the *GH1* locus (hg38) associating with adult height. The missense variant, chr17:63918372[C], is labelled as a diamond and shown in purple, and other variants are colored according to correlation ($r^2$) with that marker (legend at top-right). $-\log_{10}$ P-values are shown along the left y-axis and correspond to the variants depicted in the plot. The right y-axis shows calculated recombination rates at the chromosomal location, plotted as a solid grey line.

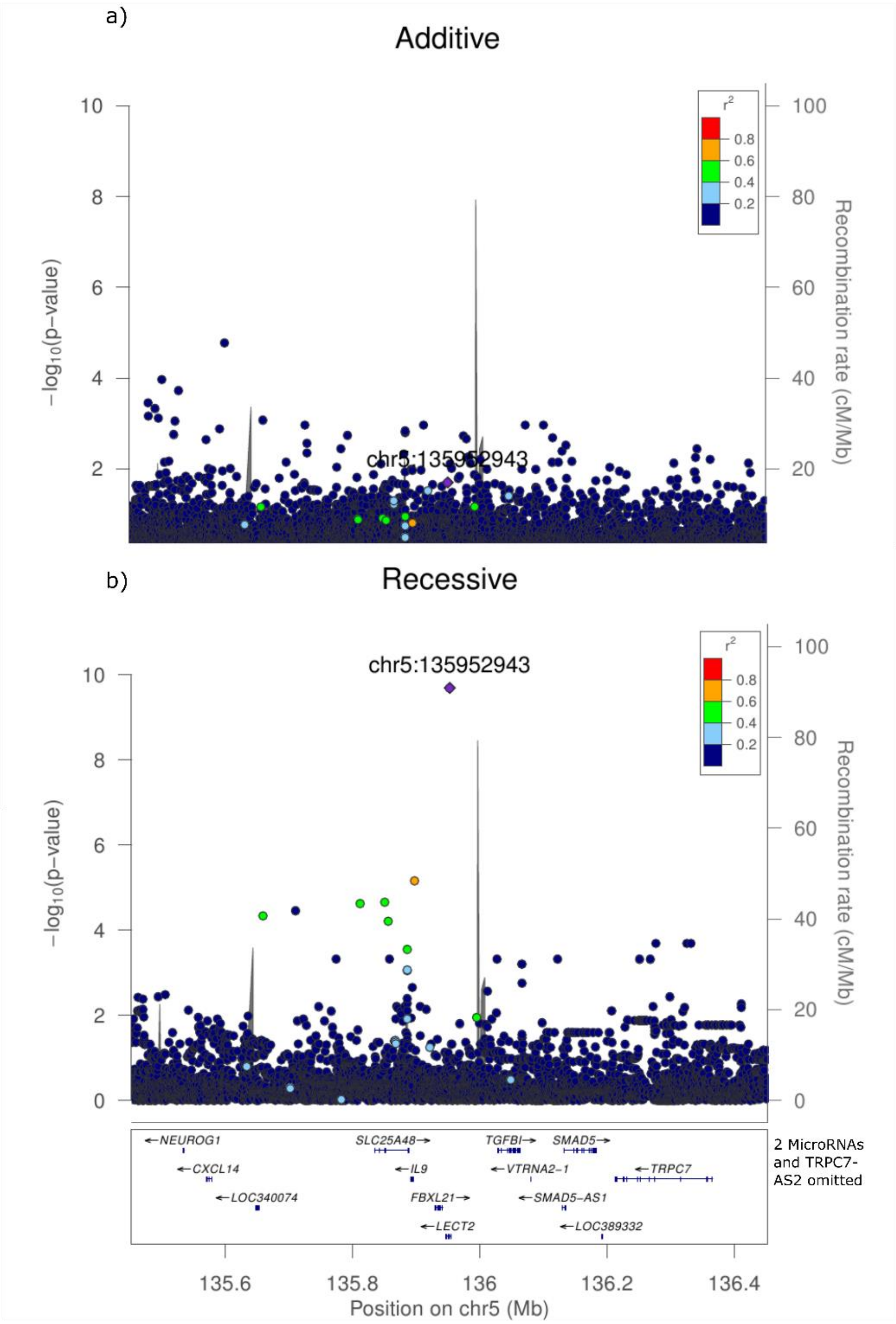


**Supplementary Fig. 12.**

Locus plots depicting variants at the *LECT2* locus (hg38) associating with adult height using the (a) additive, and (b) recessive model. Genome-wide significance is observed under the recessive model, but not under the additive model. In both plots, the leading variant under the recessive model, rs62623707[G], is labelled as a diamond and shown in purple, and other variants are colored according to correlation ($r^2$) with this marker (legend at top-right). -$\log_{10}$ P-values are shown along the left y-axis and correspond to the variants depicted in the plot. The right y-axis shows calculated recombination rates at the chromosomal location, plotted as a solid grey line.

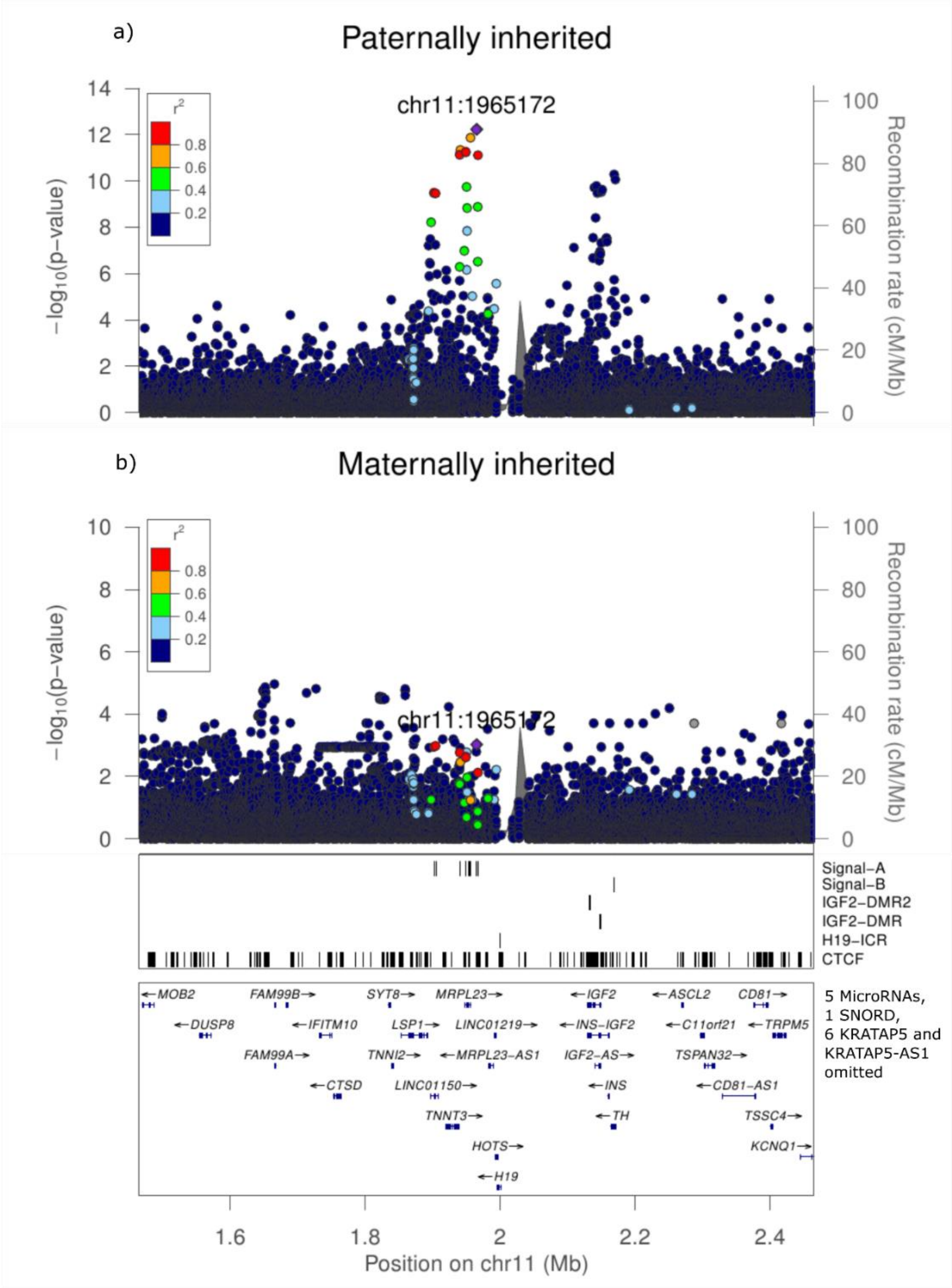


**Supplementary Fig. 13.**

Locus plot depicting rs147239461[T] at 11p15.5 (hg38) associating with adult height using the (a) paternal and (b) maternal models. Genome-wide significance is observed for rs147239461[T] when paternally inherited but not under the maternal model. rs147239461[T] is labelled as a diamond and shown in purple and other variants are colored according to correlation ($r^2$). -$\log_{10}$ P-values are shown along the left y-axis and correspond to the variants depicted in the plot. The right y-axis shows calculated recombination rates at the chromosomal location, plotted as a solid grey line.

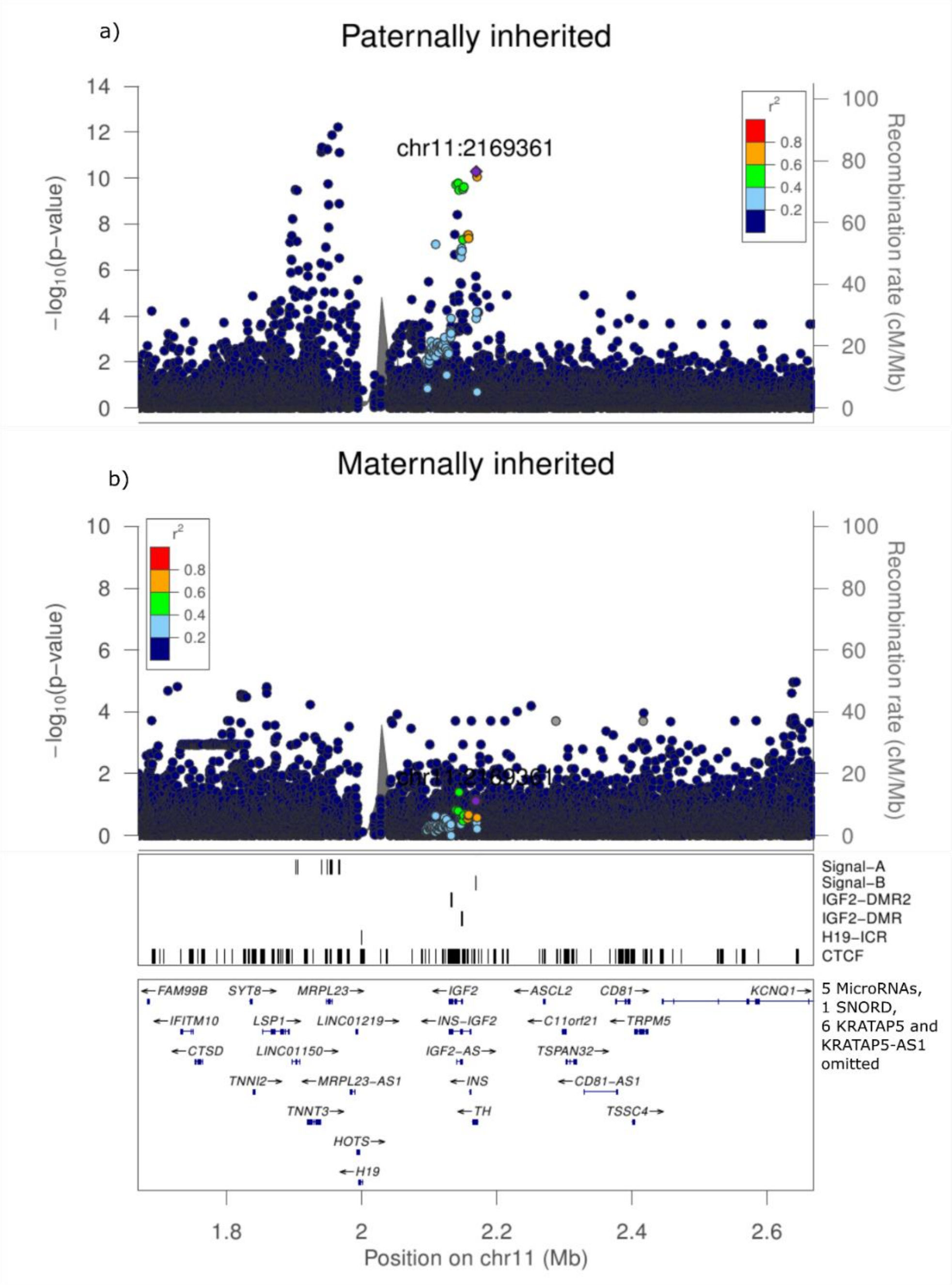


**Supplementary Fig. 14.**

Locus plot depicting rs7482510[G] at 11p15.5 (hg38) associating with adult height using the (a) paternal and (b) maternal models. Genome-wide significance is observed for rs7482510[G] when paternally inherited but not under the maternal model. rs7482510[G] is labelled as a diamond and shown in purple and other variants are colored according to correlation ($r^2$). $-\log_{10}$ P-values are shown along the left y-axis and correspond to the variants depicted in the plot. The right y-axis shows calculated recombination rates at the chromosomal location, plotted as a solid grey line.

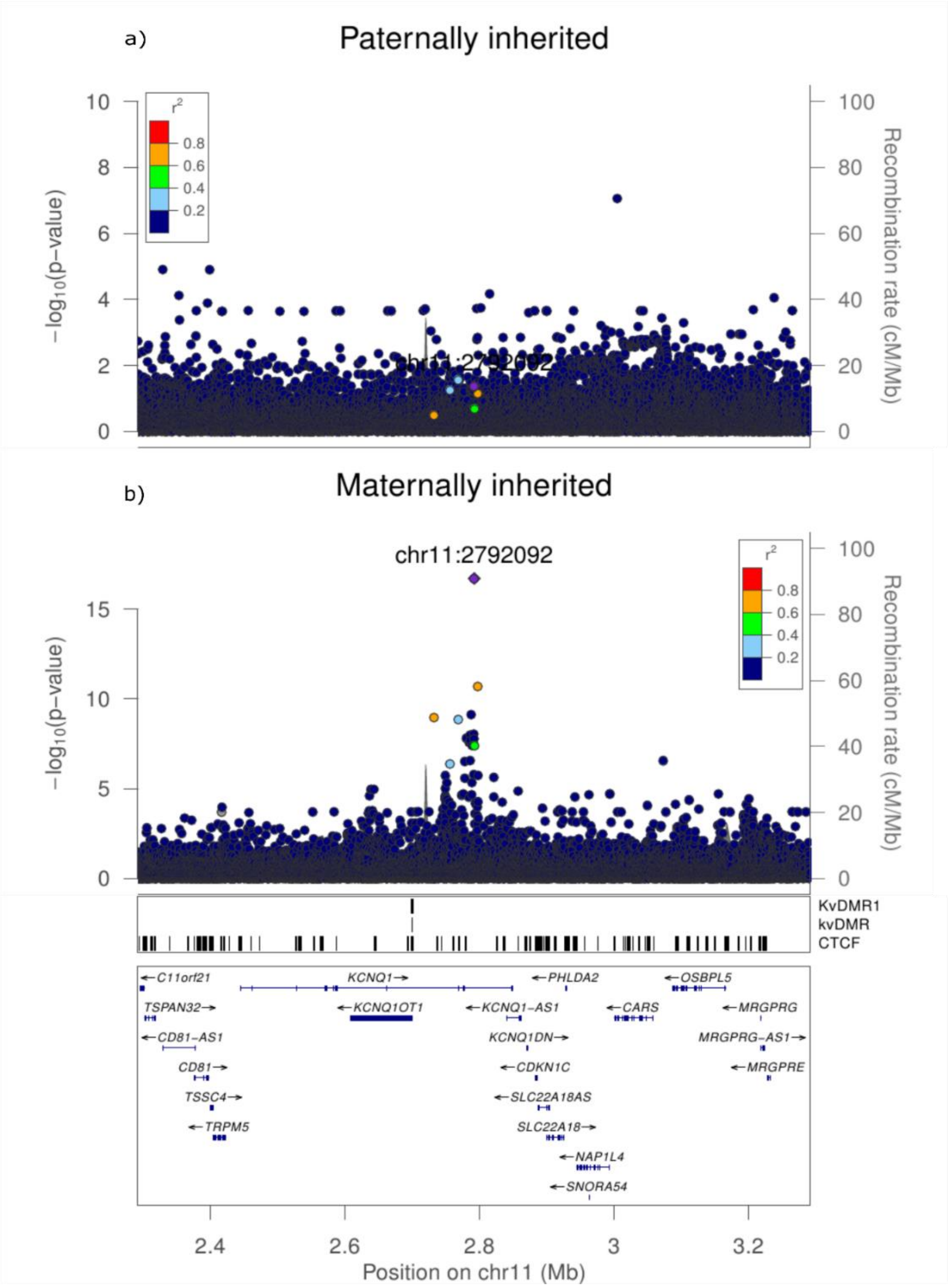


**Supplementary Fig. 15.**

Locus plot depicting rs143840904[T] at the *KCNQ1* locus (hg38) associating with adult height using the (a) paternal and (b) maternal models. Genome-wide significance is observed for rs143840904[T] when maternally inherited but not under the paternal model. rs143840904[T] is labelled as a diamond and shown in purple and other variants are colored according to correlation ($r^2$). -$\log_{10}$ P-values are shown along the left y-axis and correspond to the variants depicted in the plot. The right y-axis shows calculated recombination rates at the chromosomal location, plotted as a solid grey line.

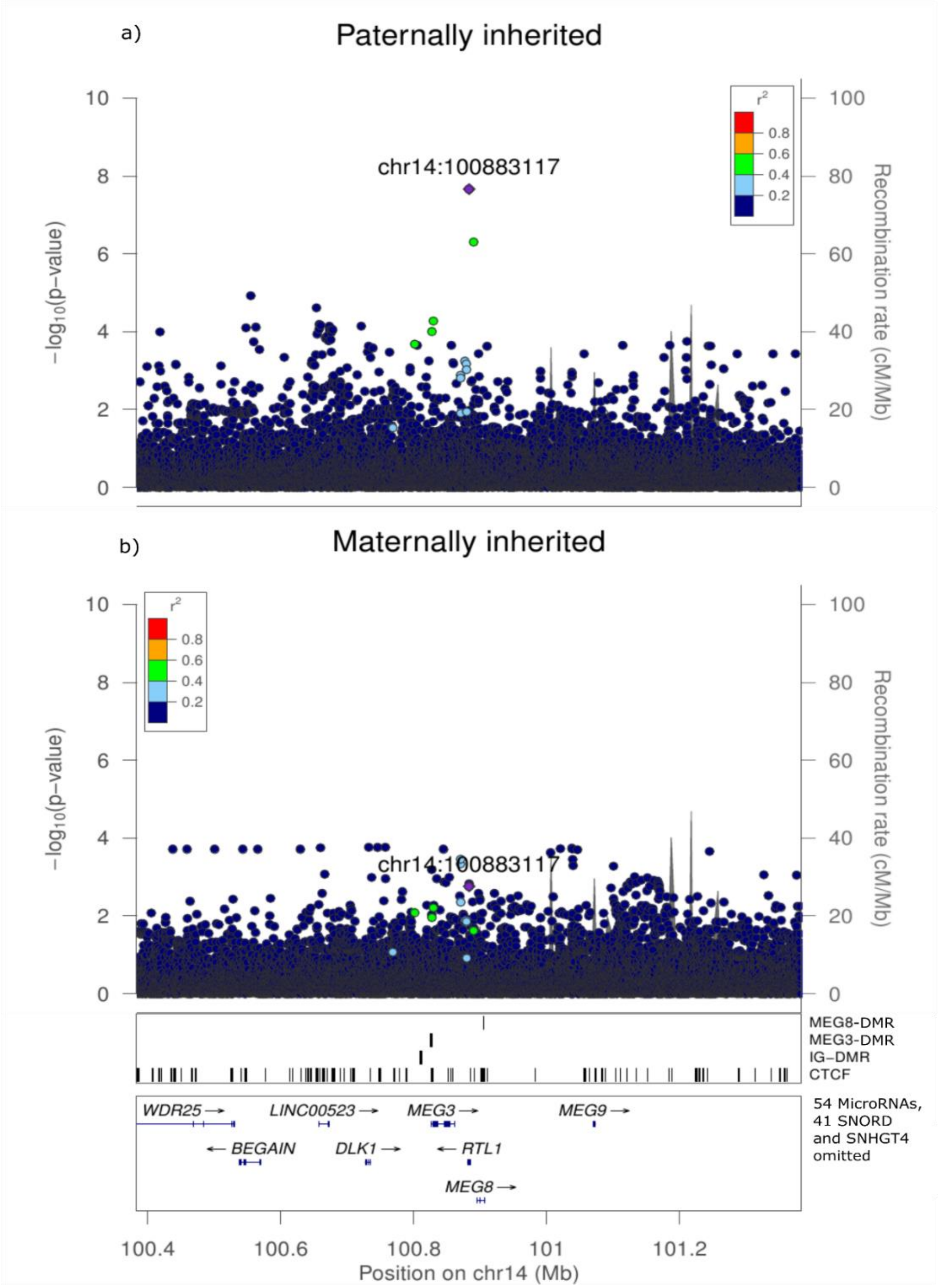


**Supplementary Fig. 16.**

Locus plot depicting variants at the *RTL1* locus (hg38) associating with adult height using the (a) paternal and (b) maternal models. Genome-wide significance is observed under the paternal model, but not under the maternal model. In all plots, the leading variant under the paternal model, rs41286560[T], is labelled and shown in purple, and other variants are colored according to correlation ($r^2$) with that marker. -$\log_{10}$ P-values are shown along the left y-axis and correspond to the variants depicted in the plot. The right y-axis shows calculated recombination rates at the chromosomal location, plotted as a solid grey line.

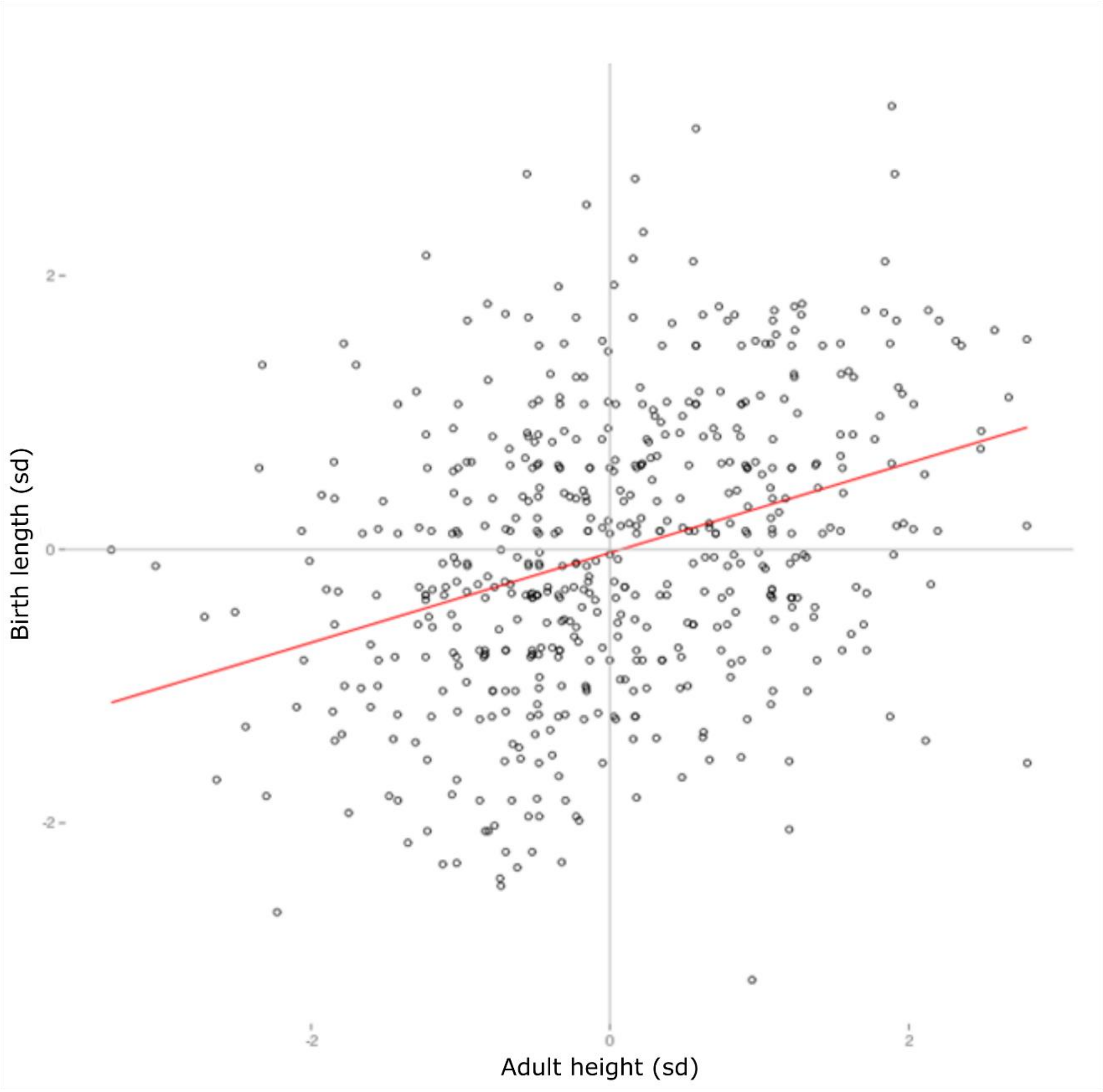


**Supplementary Fig. 17.**

Scatter plot showing adult height measurements (sd) (x-axis) and birth length measurements (sd) (y-axis) for 539 Icelanders. The red line, y = -0.025 + 0.33x +e, represents a linear regression with $R^2$ = 0.11

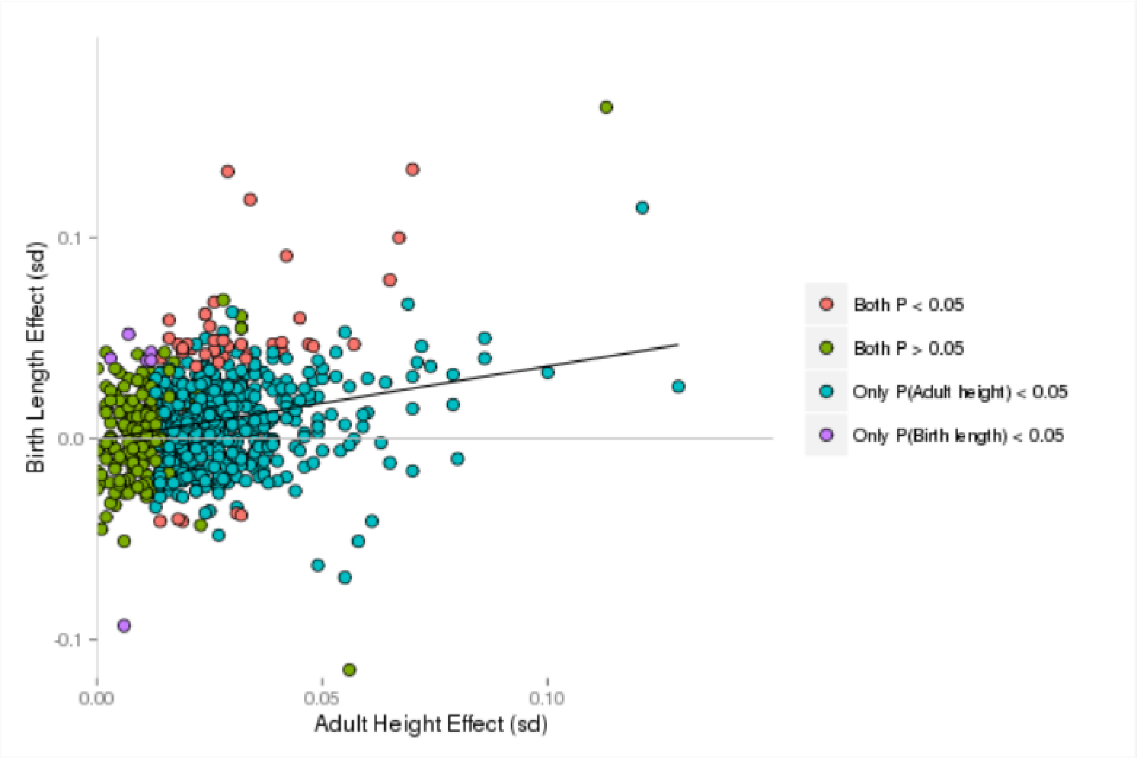


**Supplementary Fig. 18.**

Scatter plot showing how the 697 variants reported by GIANT[1] effect adult height (x-axis) and length at birth (y-axis) in our data. The effect allele (minor or major) of each variant was concluded to be the one that has a positive effect on adult height in our data. The black solid line, $y = -0.0006 + 0.37x + e$, represents weighted linear regression with $R^2 = 0.05$ using MAF(1-MAF) as weights. Different colors of points represent their *P*-values. Red points represent variants that associate with both phenotypes with $P<0.05$, green points represent variants that associate with both phenotypes with $P>0.05$, blue points represent variants that associate only with adult height with $P<0.05$ and purple points represent variants that associate only with birth length with $P<0.05$.

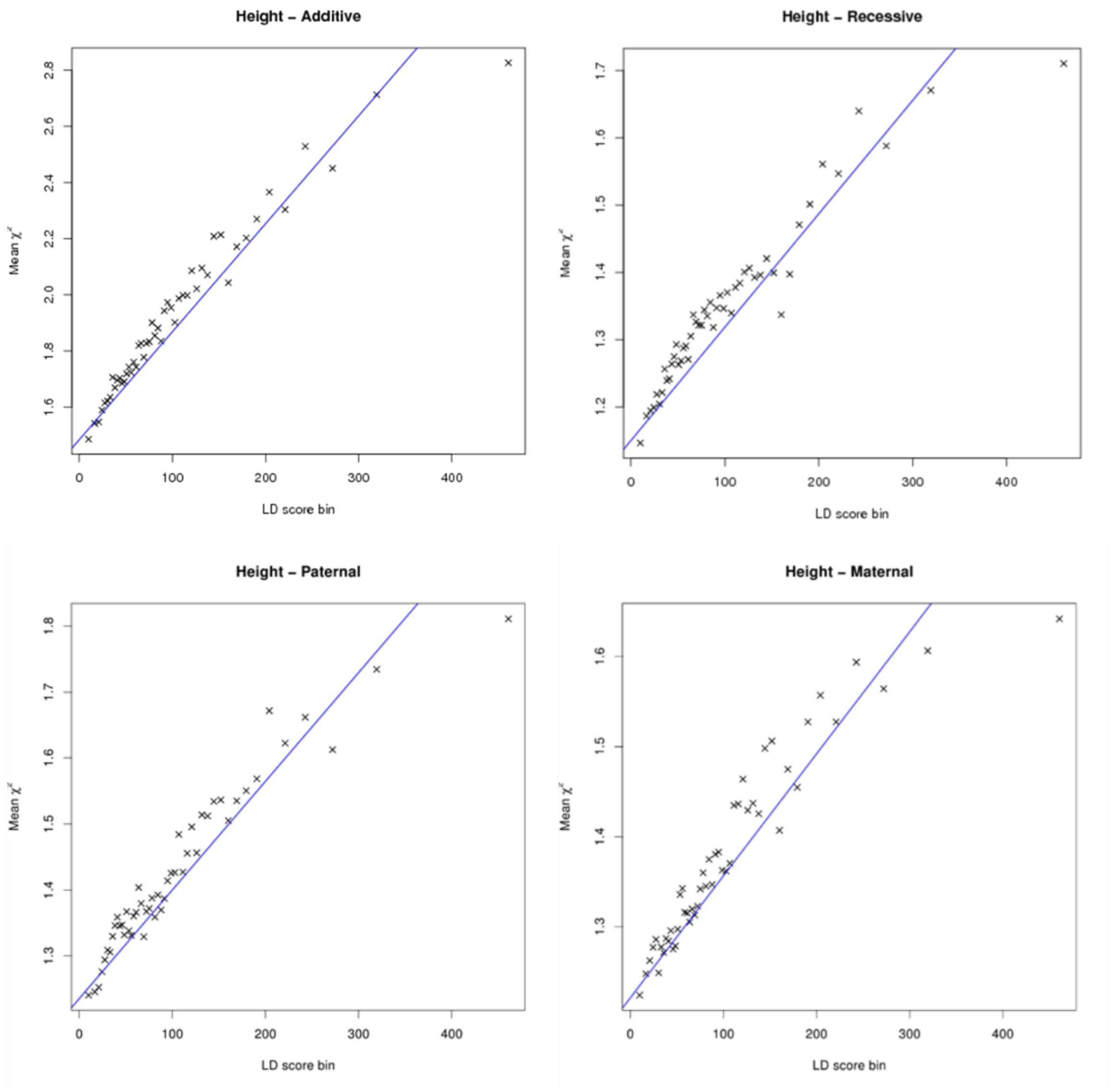


**Supplementary Fig. 19.**

LD score regression plot for height, assuming an additive, recessive, paternal and maternal genotypic model respectively. Each point represents an LD score quantile. The x-axis coordinates represent the mean LD score of a quantile and the y-axis the mean $\chi^2$ statistic of the quantile. The blue line is the LD score regression line.

**Supplementary Table 1.**

Frequency of height associated phenotypes in the OMIM database*. Tabulated is the frequency of the phenotypic term "Abnormality of body height" and its descendant terms two subclasses down in the human phenotype ontology. Only phenotypic terms that are a typical feature of a OMIM disease were regarded (typical = occourring in > 50 % of patients).

| Phenotypic abnormality | HPO ID** | #OMIM disease linked to HPO ID | OMIM disease linked to HPO ID (%) | #Genes linked to HPO ID | Genes linked to HPO ID (%) |
|---|---|---|---|---|---|
| Abnormality of body height | HP:0000002 | 603 | 13.12 | 802 | 17.45 |
| - Short stature | HP:0004322 | 560 | 12.19 | 743 | 16.17 |
| -- Disproportionate short stature | HP:0003498 | 93 | 2.04 | 96 | 2.09 |
| -- Proportionate short stature | HP:0003508 | 56 | 1.22 | 70 | 1.52 |
| -- Birth length less than 3rd percentile | HP:0003561 | 9 | 0.2 | 10 | 0.22 |
| -- Pituitary dwarfism | HP:0000839 | 6 | 0.13 | 5 | 0.11 |
| -- Asymmetric short stature | HP:0008929 | - | - | - | - |
| - Tall stature | HP:0000098 | 50 | 1.09 | 84 | 1.83 |
| -- Overgrowth | HP:0001548 | 13 | 0.28 | 18 | 0.39 |
| -- Disproportionate tall stature | HP:0001519 | 12 | 0.26 | 15 | 0.33 |
| -- Slender build | HP:0001533 | 7 | 0.15 | 10 | 0.22 |
| -- Birth length greater than 97th percentile | HP:0003517 | 4 | 0.87 | 6 | 0.13 |
| -- Proportionate tall stature | HP:0011407 | - | - | - | - |
| - Abnormal upper to lower segment ratio | HP:0012772 | 1 | 0.22 | 1 | 0.22 |
| -- Increased upper to lower segment ratio | HP:0012774 | 1 | 0.22 | 1 | 0.22 |
| -- Reduced upper to lower segment ratio | HP:0012773 | - | - | - | - |

*downloaded 17/2/2016: http://compbio.charite.de/jenkins/job/hpo.annotations.monthly/lastSuccessfulBuild/artifact/annotation/OMIM_TYPICAL_FEATURES_diseases_to_genes_to_phenotypes.txt, Additionally, HPO IDs were mapped to all its the ancestor classes as defined in the HPO OBO file to facillitate counting (hp.obo; format-versioqn: 1.2; data-version: releases/2016-01-13).

**HPO ID = human phenotype ontology (HPO) identifier for a phenotypic term as defined in the HPO OBO file (hp.obo; format-versioqn: 1.2; data-version: releases/2016-01-13).

**Supplementary Table 2.**

Year of birth and sex of imputed and family imputed individuals with adult height measurements.

| | N | YOB (mean ± sd) | Height (mean in cm) | Height (sd in cm) |
|---|---|---|---|---|
| **Male** | 32,805 | 1947 ± 19 | 178.8 | 6.9 |
| **Female** | 56,030 | 1948 ± 19 | 165.6 | 6.3 |

**Supplementary Table 3.**

Disease and phenotypic information from OMIM of monogenic growth defects linked to genes harboring novel coding variants associated with height.

| Gene | Disease Name | OMIM ID | Inheritance | HPO ID | HPO Term | Description |
|---|---|---|---|---|---|---|
| *ACAN* | SPONDYLOEPIPHYSEAL DYSPLASIA, KIMBERLEY TYPE | 608361 | Autosomal dominant | HP:0004322 | Short stature | Aggrecan/versican proteoglycan family |
| | SPONDYLOEPIPHYSEAL DYSPLASIA, KIMBERLEY TYPE | 608361 | Autosomal dominant | HP:0003508 | Proportionate short stature | |
| | OSTEOCHONDRITIS DISSECANS, SHORT STATURE | 165800 | Autosomal dominant | HP:0004322 | Short stature | |
| | SPONDYLOEPIMETAPHYSEAL DYSPLASIA, AGGRECAN TYPE | 612813 | Autosomal recessive | HP:0008905 | Rhizomelia | |
| *ADAMTS10* | WEILL-MARCHESANI SYNDROME, AUTOSOMAL RECESSIVE | 277600 | Autosomal recessive | HP:0003508 | Proportionate short stature | Metalloprotease. ADAMTS protein family |
| | ECTOPIA LENTIS 2, ISOLATED | 225100 | Autosomal recessive | HP:0001519 | - | |
| *ADAMTS17* | WEILL-MARCHESANI-LIKE SYNDROME | 613195 | Autosomal recessive | HP:0004322 | Short stature | Metalloprotease. ADAMTS protein family |
| *ADAMTSL4* | ECTOPIA LENTIS WITH ECTOPIA OF PUPIL | 225200 | Autosomal recessive | HP:0001519 | - | Metalloprotease. ADAMTS-like protein family |
| *GH1* | ISOLATED GROWTH HORMONE DEFICIENCY, TYPE II | 173100 | Autosomal dominant | HP:0000839 | Pituitary dwarfism | Growth hormone 1 |
| | ISOLATED GROWTH HORMONE DEFICIENCY, TYPE II | 173100 | Autosomal dominant | HP:0003510 | Severe short stature | |
| | PITUITARY DWARFISM I | 262400 | Autosomal recessive | HP:0000839 | Pituitary dwarfism | |
| | PITUITARY DWARFISM I | 262400 | Autosomal recessive | HP:0003510 | Severe short stature | |
| | PITUITARY DWARFISM IV | 262650 | Autosomal recessive | HP:0000839 | Pituitary dwarfism | |
| | ISOLATED GROWTH HORMONE DEFICIENCY, TYPE IB | 612781 | Autosomal recessive | HP:0004322 | Short stature | |
| *LECT2* | - | - | - | - | - | Chemotactic factor to neutrophils |
| *NPR2* | ACROMESOMELIC DYSPLASIA, MAROTEAUX TYPE | 602875 | Autosomal recessive | HP:0003498 | Disproportionate short stature | Natriuretic peptide receptor 2 |
| | ACROMESOMELIC DYSPLASIA, MAROTEAUX TYPE | 602875 | Autosomal recessive | HP:0004322 | Short stature | |
| *RTL1* | - | - | - | - | - | Retrotransposon-like-1/paternally expressed-11 |
| *TET1* | - | - | - | - | - | Leukemia associated protein |
| *ZFAT* | - | - | - | - | - | Transcriptional regulator involved in apoptosis and cell survival |

**Supplementary Table 4.**

Frequency and conservation information of novel variants associated with height.

| | | | | | ExAC MAF(%)[a] | | | | |
|---|---|---|---|---|---|---|---|---|---|
| Marker | Position (hg38) | Locus | Minor/Major | MAF(%) | Total | Eur.(Non-Finnish) | GERP | Coverage[b] | 1000 G MAF (%) [c] |
| - | chr1:150,553,749 | *ADAMTSL4* | C/CCAGAGCCCAGGCCTCTGGCA | 0.79 | - | - | -1.6 | 31[d] | 0.060 |
| rs62623707 | chr5:135,952,943 | *LECT2* | G/A | 5.56 | 2.98 | 4.20 | 5.6 | 48 | 1.32 |
| rs201828593 | chr8:134,610,589 | *ZFAT* | G/C | 0.69 | 0.028 | 0.048 | 5.1 | 27 | - |
| rs75596750 | chr8:134,610,608 | *ZFAT* | A/G | 0.35 | 0.048 | 0.081 | 3.9 | 27 | 0.040 |
| - | chr9:35,807,109 | *NPR2* | T/G | 0.058 | - | - | 5.7 | 41[d] | - |
| rs558226101 | chr10:68,686,650 | *TET1* | T/C | 0.13 | $8.2\times10^{-4}$ | $1.5\times10^{-3}$ | 3.5 | 27 | - |
| rs147239461 | chr11:1,965,172 | IGF2-H19 | T/G | 5.09 | - | - | -2.2 | - | 0.88 |
| rs7482510 | chr11:2,169,361 | IGF2-H19 | G/C | 16.84 | - | - | -1.0 | - | 25.02 |
| rs41286560 | chr14:100,883,117 | *RTL1* | T/G | 3.20 | 1.68 | 2.51 | 2.1 | 21 | 0.78 |
| - | chr15:88,872,939 | *ACAN* | A/G | 0.014 | - | - | 4.9 | 26[d] | - |
| rs72755233 | chr15:100,152,748 | *ADAMTS17* | A/G | 13.86 | 7.26 | 10.64 | 4.0 | 42 | 3.06 |
| - | chr17:63,918,372 | *GH1* | C/G | 0.93 | - | - | 1.4 | 53[d] | - |
| rs62621197 | chr19:8,605,262 | *ADAMTS10* | T/C | 2.40 | 3.55 | 5.18 | 2.1 | 20 | 1.72 |

[a] Information extracted from ExAC (accessed 22.09.2015) http://exac.broadinstitute.org/.
[b] Coverage for each variant is its read depth as noted in ExAC divided by 60,706 which is the total number of samples in the ExAC database.
[c] Information extracted from 1000 Genomes (accessed 26.09.2014) ftp://ftpdata.1000genomes.ebi.ac.uk/vol1/ftp/relea430_download .
[d] Coverage of the closest variant (up to 4-26 bases away) in ExAC with the same transcript ID

**Supplementary Table 5.**

Shown is the number of variants identified in the present study to associate with height and their fraction of variance explained in percentages. For computation of fraction of variance explained, we chose only one SNP representing each independent signal (Methods). δ denotes refinement gain of fraction of variance explained by the markers detected in the present study compared to their corresponding reported GIANT[1] markers.

| | Number of variants | | | | Variance explained (%) | | | |
|---|---|---|---|---|---|---|---|---|
| **Variant Class** | **Total** | **Novel** | **Refinement** | **Reported** | **Total** | **Novel** | **Refinement** | **Reported** |
| Genome-wide significant variants | 63 | 13 | 12 | 38 | 7.03 | 1.30 | 1.44 (δ=0.75) | 4.30 |
| **Impact class** | | | | | | | | |
| High impact | 1 | 1 | 0 | 0 | 0.06 | 0.06 | 0.00 | 0.00 |
| Moderate impact | 14 | 10 | 0 | 4 | 1.37 | 1.09 | 0.00 | 0.27 |
| Low impact | 48 | 2 | 12 | 34 | 5.61 | 0.15 | 1.44 (δ=0.75) | 4.03 |
| **Frequency class** | | | | | | | | |
| Rare (MAF < 0.5%) | 4 | 4 | 0 | 0 | 0.48 | 0.48 | 0.00 | 0.00 |
| Low frequency (0.5%≤ MAF ≤ 5%) | 8 | 5 | 1 | 2 | 0.77 | 0.41 | 0.13 (δ=0.09) | 0.22 |
| Common (MAF > 5%) | 51 | 4 | 11 | 36 | 5.79 | 0.42 | 1.30 (δ=0.66) | 4.07 |
| **Genetic model** | | | | | | | | |
| Additive | 58 | 9 | 12 | 37 | 6.67 | 1.06 | 1.44 (δ=0.75) | 4.18 |
| Recessive | 1 | 1 | 0 | 0 | 0.04 | 0.04 | 0.00 | 0.00 |
| Maternal | 1 | 0 | 0 | 1 | 0.12 | 0.00 | 0.00 | 0.12 |
| Paternal | 3 | 3 | 0 | 0 | 0.20 | 0.20 | 0.00 | 0.00 |

## Supplementary Table 6.

The four variants associated with height under a parent-of-origin model in the present study and 13 variants that have been previously reported to associate through parent-of-origin models with complex traits.

| Variants detected in the present study | | Variants detected in other studies | | | | | |
|---|---|---|---|---|---|---|---|
| **SNP ID** | **Positon (hg38)** | **SNP ID** | **Pos hg38** | **$r^2$** | **D'** | **PMID** | **Trait** |
| rs143840904 | chr11:2,792,092 | rs231362 | chr11:2,670,241 | <0.01 | 0.21 | 20016592 | Type 2 Diabetes |
| | | rs2237892 | chr11:2,818,521 | <0.01 | 1.00 | 20016592 | Type 2 Diabetes |
| | | rs2334499 | chr11:1,675,619 | <0.01 | 0.60 | 20016592 | Type 2 Diabetes |
| | | rs3817198 | chr11:1,887,776 | <0.01 | 0.29 | 20016592 | Breast Cancer |
| | | rs150199504 | chr11:2,793,730 | 0.064 | 0.99 | 26366551 | Height |
| | | rs143840904 | chr11:2,792,092 | 1.00 | 1.00 | 26366551 | Height |
| | | rs2075870 | chr11:2,768,789 | 0.37 | 0.78 | 26366551 | Height |
| | | rs149658560 | chr11:2,746,032 | 0.015 | 0.18 | 26366551 | Height |
| | | rs12790610 | chr11:2,773,768 | 0.023 | 0.19 | 26366551 | Height |
| | | rs67004488 | chr11:2,766,574 | 0.13 | 0.47 | 26366551 | Height |
| rs147239461 | chr11:1,965,172 | rs231362 | chr11:2,670,241 | <0.01 | 0.17 | 20016592 | Type 2 Diabetes |
| | | rs2237892 | chr11:2,818,521 | <0.01 | 0.47 | 20016592 | Type 2 Diabetes |
| | | rs2334499 | chr11:1,675,619 | <0.01 | 0.04 | 20016592 | Type 2 Diabetes |
| | | rs3817198 | chr11:1,887,776 | 0.013 | 0.74 | 20016592 | Breast Cancer |
| | | rs150199504 | chr11:2,793,730 | <0.01 | 0.1 | 26366551 | Height |
| | | rs143840904 | chr11:2,792,092 | <0.01 | 0.81 | 26366551 | Height |
| | | rs2075870 | chr11:2,768,789 | <0.01 | 0.77 | 26366551 | Height |
| | | rs149658560 | chr11:2,746,032 | <0.01 | 0.62 | 26366551 | Height |
| | | rs12790610 | chr11:2,773,768 | <0.01 | 0.67 | 26366551 | Height |
| | | rs67004488 | chr11:2,766,574 | <0.01 | 0.71 | 26366551 | Height |
| rs7482510 | chr11:2,169,361 | rs231362 | chr11:2,670,241 | <0.01 | 0.012 | 20016592 | Type 2 Diabetes |
| | | rs2237892 | chr11:2,818,521 | <0.01 | 0.54 | 20016592 | Type 2 Diabetes |
| | | rs2334499 | chr11:1,675,619 | <0.01 | 0.05 | 20016592 | Type 2 Diabetes |
| | | rs3817198 | chr11:1,887,776 | <0.01 | 0.01 | 20016592 | Breast Cancer |
| | | rs150199504 | chr11:2,793,730 | <0.01 | 0.86 | 26366551 | Height |
| | | rs143840904 | chr11:2,792,092 | <0.01 | 0.42 | 26366551 | Height |
| | | rs2075870 | chr11:2,768,789 | <0.01 | 0.30 | 26366551 | Height |
| | | rs149658560 | chr11:2,746,032 | <0.01 | 0.36 | 26366551 | Height |
| | | rs12790610 | chr11:2,773,768 | <0.01 | 0.02 | 26366551 | Height |
| | | rs67004488 | chr11:2,766,574 | <0.01 | 0.01 | 26366551 | Height |
| rs41286560 | chr14:100,883,117 | rs10144321 | chr14:100,416,068 | <0.01 | 0.16 | 25231870 | Age at Menarche |
| | | rs7141210 | chr14:100,716,133 | <0.01 | 0.20 | 25231870 | Age at Menarche |
| | | rs941576 | chr14:100,839,708 | 0.04 | 0.87 | 19966805 | Type 1 Diabetes |

**Supplementary Table 7.**

Two-way conditional analysis of the two variants at the IGF2-H19 locus that, in our data, associate with less height when paternally inherited.

| | | Variant 1 conditioned on variant 2 (Paternal model) | | | |
|---|---|---|---|---|---|
| **Variant 1** | **Variant 2** | ***P*** | ***Adj. P*** | **β(SD)** | **Adj. β(SD)** |
| rs147239461[T] | rs7482510[G] | $5.9\times10^{-13}$ | $2.2\times10^{-10}$ | -0.12 | -0.11 |
| rs7482510[G] | rs147239461[T] | $5.1\times10^{-11}$ | $1.8\times10^{-9}$ | -0.065 | -0.060 |

**Supplementary Table 8.**

Two-way conditional analysis of rs143840904 with reported Sardinian variants at the same locus under the maternal model.

| | | | Icelandic variant cond. on Sardinian variant | | | | Sardinian variant cond. on Icelandic variant | | | |
|---|---|---|---|---|---|---|---|---|---|---|
| **Iceland** | **Sardinia**[b] | **$r^2$** | ***P*** | **Adj. *P*** | **β(SD)** | **Adj. β(SD)** | ***P*** | **Adj. *P*** | **β(SD)** | **Adj. β(SD)** |
| rs143840904[a] | rs143840904 | 1.00 | $2.0\times10^{-17}$ | - | -0.26 | - | $2.0\times10^{-17}$ | - | -0.26 | - |
| | rs150199504 | 0.064 | $2.0\times 10^{-17}$ | $4.6\times10^{-15}$ | -0.26 | -0.24 | $2.4\times10^{-4}$ | $9.8\times10^{-2}$ | -0.38 | -0.18 |
| | rs2075870 | 0.37 | $2.0\times10^{-17}$ | $1.3\times10^{-09}$ | -0.26 | -0.23 | $1.4\times10^{-9}$ | 0.25 | -0.23 | -0.055 |
| | rs149658560 | 0.015 | $2.0\times10^{-17}$ | $2.2\times10^{-16}$ | -0.26 | -0.25 | $3.7\times10^{-3}$ | $5.5\times10^{-2}$ | -0.058 | -0.038 |
| | rs12790610 | 0.023 | $2.0\times10^{-17}$ | $4.0\times10^{-17}$ | -0.26 | -0.26 | 0.24 | 0.96 | -0.027 | 0.0010 |
| | rs67004488 | 0.13 | $2.0\times10^{-17}$ | $4.2\times10^{-16}$ | -0.26 | -0.26 | $1.3\times10^{-2}$ | 0.65 | -0.054 | 0.011 |
| | ***$All_{Sardinia}$ [N=5]***[c] | - | **$2.0\times10^{-17}$** | **$6.7\times10^{-10}$** | **-0.26** | **-0.24** | - | - | - | - |

[a] In the Icelandic population this variant has a significant effect on height when maternally inherited.

[b] These variants were reported to have a significant effect on height when maternally inherited in a Sardinian population [2].

[c] All the variants reported in the Sardinan population[2] excluding rs143840904.

**Supplementary Table 9.**

Simple linear regression for transmitted and untransmitted alleles of the four variants that associate with height under a parent-of-origin model in the present study.

| | | | | Transm. paternal allele | | | Transm. maternal allele | | | Untransm. pat. or mat. allele | | | | Transm vs. Untransm | |
|---|---|---|---|---|---|---|---|---|---|---|---|---|---|---|---|
| **SNV ID** | **MAF(%)** | **Gene** | **Phen**[a] | ***P*** | **β(SD)** | ***95% conf.*** | ***P*** | **β(SD)** | ***95% conf.*** | ***P*** | **β(SD)** | ***95% conf.*** | **Untr.**[b] | ***P*** | **N**[c] |
| rs147239461 | 5.1 | *IGF2-H19(A)* | AH | $1.4\times10^{-5}$ | -0.11 | (-0.162 , -0.061) | 0.30 | 0.026 | (-0.023 , 0.076) | 0.59 | 0.013 | (-0.035 , 0.062) | Pat. | $4.7\times10^{-4}$ | 29,945 |
| | | | BL | $4.6\times10^{-2}$ | -0.16 | (-0.320 , -0.003) | 0.40 | 0.067 | (-0.090,0.224) | 0.82 | 0.018 | (-0.134,0.171) | Pat. | 0.11 | 3,313 |
| rs7482510 | 16.8 | *IGF2-H19(B)* | AH | $2.8\times10^{-4}$ | -0.055 | (-0.085 , -0.026) | 0.51 | 0.010 | (-0.020 , 0.040) | 0.15 | -0.022 | (-0.051 , 0.0076) | Pat. | 0.11 | 29,948 |
| rs143840904 | 1.8 | *KCNQ1* | AH | 0.71 | -0.014 | (-0.090 , 0.062) | $3.2\times10^{-9}$ | -0.24 | (-0.322 , -0.162) | 0.81 | 0.0098 | (-0.068 , 0.088) | Mat. | $1.0\times10^{-5}$ | 39,291 |
| rs41286560 | 3.2 | *RTL1* | AH | $1.7\times10^{-5}$ | -0.14 | (-0.200 , -0.075) | 0.74 | -0.011 | (-0.077 , 0.055) | 0.85 | -0.0058 | (-0.066 , 0.054) | Pat. | $3.2\times10^{-3}$ | 29,956 |

[a] Phen: Phenotype. AH: Adult height. BL: Birth length. The variant rs147239461 also associates with birth length when paternally inherited and therefor regression is also performed for that phenotype

[b] Untr: Untransmitted. Pat: Paternal. Mat: Maternal. The variant rs143840904 associates with adult height when maternally inherited and therefore linear regression is performed for the untransmitted maternal allele. The other three associate with adult height when paternally inherited and therefore linear regression is performed for the untransmitted paternal allele.

[c] Linear regression was performed for each variant using information about N chip-typed individuals with either imputed mothers (for untransmitted maternal allele analysis) or imputed fathers (for untransmitted paternal allele analysis) (Methods).

# Supplementary Discussion

## Novel signals detected under the additive model

Nine novel height association signals were identified using the additive model. One is common (MAF>5%), four are low frequency (MAF = 0.5-5%) and four are rare (MAF < 0.5%) (Table 1). Five are in genes linked to monogenic growth defects (*NPR2, ACAN, GH1, ADAMTS10* and *ADAMTS17*) (Supplementary Table 3). The nine novel variants are shortly described below.

Two distinct missense variants in *ZFAT,* encoding a *zinc finger protein, are* associated with greater adult height; rs75596750[A] (MAF = 0.35%, p.Arg154Trp, β = 0.32 SD, $P = 8.9 \times 10^{-10}$), and rs201828593[G] (MAF = 0.69%, p.Arg160Thr, β = 0.20 SD, $P = 8.0 \times 10^{-8}$) (Table 1, Supplementary Data 5 and Supplementary Fig. 4). These two variants are only 19 base pairs (bp) apart, but the alleles associating with greater height were never observed on the same chromosome ($r^2 = 0.00003$).

A rare missense variant in *TET1*, rs558226101[T] (MAF = 0.13%, p.Arg1783Trp), is associated with greater adult height (β = 0.48 SD, $P = 1.6 \times 10^{-8}$) (Table 1, Supplementary Data 5 and Supplementary Fig. 3). To our knowledge, *TET1* have not been reported to associate with height in humans, but *Tet1* paternal knockout mice suffer fetal and postnatal growth defects [3].

A rare missense variant, (chr9:35807109[T], MAF = 0.058%, p.Gly869Val) in *NPR2,* encoding the natriuretic peptide receptor 2, has a large effect on adult height (β = -1.53 SD, $P = 3.6 \times 10^{-31}$) and was not observed outside of Iceland (Table 1, Supplementary Data 5, Supplementary Table 4 and Supplementary Fig. 5). This amounts to heterozygous carriers being 10.1 cm shorter than the population average. Other mutations in *NPR2* are known to cause Maroteaux-type acromelic dysplasia under a recessive mode of inheritance [4] (Supplementary Table 3). The observed

association with less height is consistent with the reported heterozygous effect on height (-1.8 SD) in relatives of a proband with this dysplasia [5] and findings in individuals with idiopathic short stature [6,7]. We observed no homozygous carriers of p.Gly869Val in our data.

A very rare missense variant (chr15:88872939[A], MAF = 0.014%, p.Cys2416Tyr) in the gene *ACAN* is associated with substantially less height ($\beta$ = -1.61 SD, $P = 1.2 \times 10^{-9}$) (Table 1, Supplementary Data 5 and Supplementary Fig. 6). All 28 carriers of this rare variant were clustered within a single five-generation pedigree (Supplementary Fig. 7). Detection and imputation of this variant was facilitated by enriching for phenotypic extremes in the WGS set (N = 8,453), where eight heterozygous carriers were observed but only two would have been expected by chance given the allele frequency of 0.014% based on imputation. Previously identified sequence variants in *ACAN* cause Mendelian conditions that affect height through both autosomal dominant and recessive inheritance (Supplementary Table 3).

A frameshift insertion (chr1:150553749[C], MAF = 0.79%, p.Gln256ProfsTer38) in *ADAMTSL4* associates with less height ($\beta$ = -0.20 SD, $P = 2.0 \times 10^{-8}$) (Table 1, Supplementary Data 5 and Supplementary Fig. 8). Ectopia lentis has been described in homozygous carriers for this variant, which represents an ancient European founder mutation[8].

One of the novel height signals is represented by a common missense variant rs72755233[A] (MAF = 13.86%; p.Thr446Ile) in *ADAMTS17* that is associated with decreased adult height ($\beta$ = -0.098 SD, $P = 8.7 \times 10^{-27}$) (Table 1 and Supplementary Data 5). This variant is not strongly correlated ( all $r^2 < 0.40$) with any other variant in our data (Supplementary Fig. 9) or the 1000 Genomes data, it is not present in HapMap (1000 Genomes, ftp://ftpdata.1000genomes.ebi.ac.uk/vol1/ftp/relea430_download; accessed 26.09.2014,

HapMap, hapmap.ncbi.nlm.nih.gov/cgi-perl/gbrowse/hapmap28_B36; accessed 26.08.2015), and was not tested in the GIANT study [1].

The low-frequency missense variant rs62621197[T] (MAF = 2.40%, p.Arg62Gln) in *ADAMTS10* associates with less adult height (β = -0.16 SD, $P = 7.3 \times 10^{-14}$) (Table 1 and Supplementary Fig. 10). Both *ADAMTS10*, and *ADAMTS17* (which yielded a novel height-associated common missense variant) are members of the ADAMTS family of proteins. Rare sequence variants in *ADAMTS10* and *ADAMTS17* cause monogenic growth defects under a recessive model [9] (Supplementary Table 3).

The low-frequency missense variant (chr17:63918372[C], p.Leu49Val, MAF = 0.93%) in *GH1,* encoding growth hormone 1, was associated with less height (β = -0.26 SD, $P = 1.0 \times 10^{-15}$) (Table 1, Supplementary Data 5 and Supplementary Fig. 11). Carriers of this variant in Iceland are 1.7 cm shorter than the population average. Mutations in *GH1* cause growth hormone deficiencies (characterized by short stature) through both dominant and recessive modes of inheritance [10] (Supplementary Table 3). The eight imputed homozygotes with height measurements were on average 4.4 cm shorter than the population average, indicating a dosage effect. To our knowledge, p.Leu49Val has not been observed in population catalogs of sequence variation despite good coverage (ExAC, http://exac.broadinstitute.org/; accessed 22.09.2015), but is carried by one in 56 Icelanders (Supplementary Table 4). This is an example of a founder effect similar to that reported for a missense variant in the gene *GHR* in Sardinina [2] (rs121909358; $MAF_{sardinia}$ = 0.87 %, Allele count in Europeans ExAC = 1/73,354).

## Novel signal detected under the recessive model

One novel signal was detected under a recessive model. The association is represented by a missense variant in *LECT2*, rs62623707[G] (homozygous genotype frequency = 0.31 %;

p.Ile24Thr) (Table 1, Supplementary Data 5 and Supplementary Fig. 12). An association with height would not have been detected under the additive model ($P_{add}$ = 0.020).

## Supplementary References


1. Wood, A. R. *et al.* Defining the role of common variation in the genomic and biological architecture of adult human height. *Nat. Genet.* **46,** 1173–1186 (2014).
2. Zoledziewska, M. *et al.* Height-reducing variants and selection for short stature in Sardinia. *Nat. Genet.* **47,** 1352–1356 (2015).
3. Yamaguchi, S., Shen, L., Liu, Y., Sendler, D. & Zhang, Y. Role of Tet1 in erasure of genomic imprinting. *Nature* **504,** 460–464 (2013).
4. Bartels, C. F. *et al.* Mutations in the transmembrane natriuretic peptide receptor NPR-B impair skeletal growth and cause acromesomelic dysplasia, type Maroteaux. *Am. J. Hum. Genet.* **75,** 27–34 (2004).
5. Olney, R. C. *et al.* Heterozygous mutations in natriuretic peptide receptor-B (NPR2) are associated with short stature. *J. Clin. Endocrinol. Metab.* **91,** 1229–1232 (2006).
6. Vasques, G. A. *et al.* Heterozygous mutations in natriuretic peptide receptor-B (NPR2) gene as a cause of short stature in patients initially classified as idiopathic short stature. *J. Clin. Endocrinol. Metab.* **98,** (2013).
7. Amano, N. *et al.* Identification and Functional Characterization of Two Novel NPR2 Mutations in Japanese Patients with Short Stature. *J. Clin. Endocrinol. Metab.* **99,** E713–E718 (2014).
8. Neuhann, T. M. *et al.* ADAMTSL4-associated isolated ectopia lentis: Further patients, novel mutations and a detailed phenotype description. *Am. J. Med. Genet. Part A* **167,** 2376–2381 (2015).
9. Morales, J. *et al.* Homozygous Mutations in ADAMTS10 and ADAMTS17 Cause Lenticular Myopia, Ectopia Lentis, Glaucoma, Spherophakia, and Short Stature. *Am. J. Hum. Genet.*

**85,** 558–568 (2009).

10. Alatzoglou, K. S. & Dattani, M. T. Genetic causes and treatment of isolated growth hormone deficiency-an update. *Nat. Rev. Endocrinol.* **6,** 562–576 (2010).
